# Reduced Right Frontal Fractional Anisotropy Correlated with Early Elevated Plasma LDL Levels in Obese Young Adults

**DOI:** 10.1371/journal.pone.0108180

**Published:** 2014-10-03

**Authors:** Baohui Lou, Min Chen, Xiaojie Luo, Yongming Dai

**Affiliations:** 1 Graduate School of Peking Union Medical College, Beijing, China; 2 Beijing Institute of Geriatrics, Beijing Hospital, Beijing, China; 3 Department of Radiology, Beijing Hospital, Beijing, China; 4 Philips Healthcare, Shanghai, China; Nathan Kline Institute and New York University School of Medicine, United States of America

## Abstract

**Objective:**

To investigate the underlying physiological mechanisms of the structural differences in gray matter (GM) and white matter (WM) associated with obesity in young Chinese adults.

**Materials and Methods:**

A total of 49 right-handed obese or overweight (n = 22, mean age 31.72±8.04 years) and normal weight (n = 27, mean age 29.04±7.32 years) Han Chinese individuals were recruited. All participants underwent voxel-based morphometry analysis of T1-weighted MRI and tract-based spatial statistics analysis of diffusion tensor imaging. Partial correlation analysis was performed between the physiological data obtained and the abnormal structural alterations.

**Results:**

In the OO group, GM atrophy occurred in the left prefrontal cortex, bilateral cingulate gyrus, and the right temporal lobe, while enlargement was observed in the bilateral putamen. WM atrophy was observed predominantly in the regions that regulate food intake, such as the bilateral basal ganglia, the right amygdala, and the left insula. The OO group exhibited lower fractional anisotropy (FA) in bilateral frontal corticospinal tracts and the right brainstem. Significant negative correlations were observed between FA values of those three clusters and BMI, and waist circumference, while the volume of bilateral putamen positively correlated with both BMI and waist circumference. High plasma LDL levels were correlated with low FA values in the right frontal corticospinal tract. Interestingly, the negative correlation was limited to male participants.

**Conclusions:**

Obesity-related alterations of GM and WM volumes were observed predominantly in food reward circuit, which may motivate abnormal dietary intake. Further, early elevated plasma LDL might contribute to low right frontal FA values of male adults, which requires further demonstration by larger-scale and longitudinal studies.

## Introduction

Obesity has become a major public health concern worldwide [1]. According to the epidemiological data, excessive body weight (overweight) and obesity had 42.6% prevalence among Chinese adults in 2010, with the urban population showing a higher prevalence than the rural one [2]. In China, this increased prevalence of overweight and obesity was considered to be related to the modifications in both diet habits and physical activities [3]. The higher fat content in animal-derived foods conspired with increasingly sedentary lifestyle resulted in abnormally high accumulation of both plasma lipids and adipose tissues (body fat) [4].

As an important constituent of cellular membranes and other structures such as myelin, cholesterol is considered as an essential component of brain structure. Abnormal brain cholesterol metabolism is associated with neurodegenerative diseases such as Alzheimer's disease (AD) and Parkinson's disease (PD) [5]. High levels of plasma cholesterol have been linked to increased expression of AD-related pathology, and studies employing statins as cholesterol-lowering drugs have exhibited their protective effect against AD [6], [7]. The findings of these studies suggested that high plasma cholesterol levels are associated with abnormal brain structures in individuals with the aforementioned neurodegenerative diseases; however, the mechanisms underlying this correlation remain unclear.

Previous studies suggest that obesity would increase the risk of neurodegenerative disorders [8], [9]. Accumulating evidence suggested that obesity was associated with gray matter (GM) atrophy in certain brain areas [10]. Negative correlation was observed between body mass index (BMI), as one measure of excess adiposity, and GM volumes in specific brain regions [11], [12]. Moreover, longitudinal analysis demonstrated that higher BMI was related to decline in the temporal, occipital, and frontal GM volumes [11], [13]. In addition, inconsistent findings were obtained from previous studies on obesity-associated alterations of white matter (WM) volume. Several of these studies reported greater WM volume in the striatum [10], temporal gyrus, parahippocampal gyrus, brainstem, and cerebellum [14] in obese versus lean individuals, whereas some other studies showed negative correlation between BMI and WM volumes in the orbital frontal cortex, anterior cingulate gyrus, medial temporal lobe, and subcortical regions [15]. A recent study also demonstrated that obese individuals possessed smaller WM volume in the limbic regions (e.g., the insula and amygdala) and within the superior and middle temporal lobes [16]. Diffusion tensor imaging (DTI) has provided little information about the microstructural alterations of WM associated with obesity, although fractional anisotropy (FA) generated from DTI was most commonly applied for assessing WM integrity. The negative association between BMI and FA was revealed in Stanek et al's study [17]. FA reductions were revealed in the corticospinal tracts, mamillary bodies, optic radiations, corpus callosum (CC), and the right inferior occipito-frontal fascicle in obese individuals [16], while obesity-associated reductions in FA were observed to occur mainly in the midbrain and brainstem tracts in another study [18]. Moreover, a significant negative correlation between BMI and FA was observed in the CC in females rather than males [19].

Despite the observations of diverse structural abnormalities in the GM and WM of obese individuals, the physiological mechanisms underlying these alterations remained ambiguous. Considering the influence of plasma cholesterol level on brain structure in neurodegenerative diseases, it was hypothesized that the high plasma lipid level associated with obesity might contribute to the structural abnormalities detected in the brains of obese or overweight individuals. In the present study, voxel-based morphometry (VBM) and tract-based spatial statistics (TBSS) were applied to compare the alterations in the brain structures of Chinese obese or overweight (OO) young adults and their normal weight (NW) counterparts, and to determine whether elevated plasma lipid values correlate with these alterations.

## Materials and Methods

### Sample selection

A total of 49 right-handed Han Chinese subjects, 27 of which were NW (15 men and 12 women, mean age 29.04±7.32 years, BMI 21.54±2.06 kg/m^2^) and 22 were OO (12 men and 10 women, mean age 31.72±8.04 years, BMI 31.44±3.34 kg/m^2^), were recruited from the physical examination center of Beijing Hospital (China). The participants were categorized as NW (BMI 18.5–23.9 kg/m^2^), overweight (BMI 24.0–27.9 kg/m^2^), and obese (BMI≥28.0 kg/m^2^), according to the Chinese criteria of obesity [20]. All participants were healthy, as determined by the results of their physical examinations and blood tests. Individuals with diabetes, hyperglycemia, hypertension, those on any medications, and those with a history of metabolic, genetic, or neurological disorders were excluded from the study. All the selected subjects were evaluated using the Hospital Anxiety and Depression Scale (HADS). A cutoff score of 11 was applied to each scale of HADS [21]. This study was approved by the Medical Research Ethics Committee of Beijing Hospital. Written informed consent was obtained from each participant and submitted to the ethics committee of the hospital.

After a 12-h overnight fast and prior to magnetic resonance imaging (MRI) scanning, the participants underwent blood tests to measure their glucose, triglyceride, HDL, LDL, and total cholesterol levels. Data regarding the heights, weights, waist circumference, and blood pressure of the participants were collected. BMI was defined as the weight in kilograms divided by the square of height in meters. Waist circumference was measured with a tape centered at the umbilicus.

### MRI data acquisition

Data was acquired using a 3.0T magnetic resonance scanner with an 8-channel head coil. Foam padding was applied to minimize head movement. Preliminary conventional T2-weighted images were collected in order to exclude individuals with potential neurological diseases.

DTI data were acquired by employing a single-shot echo planar imaging sequence. A total of 60 continuous axial slices were obtained with diffusion sensitizing gradients applied along 32 non-collinear directions (b = 1000 s/mm^2^). The parameters were as follows: repetition time (TR) = 9226 ms, echo time (TE) = 83 ms, matrix size = 256 mm×256 mm, number of excitations (NEX) = 1, reconstruction matrix size = 128 mm×128 mm, field of view (FOV) = 240 mm×240 mm, and slice thickness = 2 mm with no interslice gap.

High-resolution T1-weighted images were acquired with a three-dimensional balanced turbo field echo sequence. The parameters were as follows: TR = 7.4 ms, TE = 3 ms, flip angle = 8°, FOV = 240 mm×240 mm, matrix size = 256 mm×256 mm, NEX = 1, voxel size = 1 mm×1 mm, and slice thickness = 1 mm.

### VBM analysis

VBM was performed using diffeomorphic anatomical registration through exponentiated lie algebra algorithm (DARTEL) approach by applying the Statistical Parametric Mapping Software (SPM8, Wellcome Department of Imaging Neuroscience, University College, London, UK) running on MATLAB R2011b (The Mathworks, Sherborn, MA, USA) [22], [23]. Segmented GM, WM, and cerebrospinal fluid (CSF) images of each subject were imported to DARTEL in order to apply a nonlinear registration procedure. The segmented images were modulated with Jacobian determinants to incorporate volume modifications. These normalized modulated images were smoothed with an isotropic Gaussian kernel of 8-mm full-width-at-half-maximum. Eventually, GM, WM, and CSF volumes were calculated by applying SPM8. For statistical analysis, voxel-wise group comparisons of GM or WM volumes were implemented using two-sample *t*-test covarying for age and gender. *P*<0.05 at a cluster size of >100 voxels was considered statistically significant, after correction for false discovery rate (FDR).

### TBSS analysis

All diffusion-weighted (DW) images were preprocessed by applying the FMRIB's Diffusion Toolbox (Functional Magnetic Resonance Imaging of the Brain Analysis Group, Oxford University, UK; FMRIB Software Library [FSL] 4.0) [24]. The images were initially corrected for eddy-current-induced distortion and head motion artifacts by applying affine alignment of each DW image to *b* = 0 image. Subsequently, these images were skull-stripped using the FMRIB Brain Extraction Tool. Finally, a diffusion tensor model was fitted for each voxel using FMRIB's DTIfit to generate voxel-wise maps of FA.

The DW images were analyzed by the TBSS method [25]. The FA images of all subjects were co-registered to a template of the averaged FA images (FMRIB58_FA) in Montreal Neurological Institute space using a nonlinear registration algorithm implemented in FNIRT (FMRIB's nonlinear registration tool) [26]. A mean FA image and mean FA skeleton of the WM tracts were subsequently created. The aligned FA images of each subject were then projected onto the mean FA skeleton to create a skeletonized FA map. Voxel-wise statistical analysis of skeletonized FA data was performed using a permutation-based inference tool for nonparametric statistics (‘randomize’ program, part of FSL). A mean FA value of 0.2 was set as a threshold for the mean FA skeleton in order to exclude peripheral tracts and preserve the main fiber tracks. The number of random permutations was set to 10,000. Two-sample *t*-tests were used for intergroup comparisons, controlling age and gender as no-interest regressors. The statistical threshold was set at *P*<0.05 (corrected for multiple comparisons), applying FSL's permutation-testing tool's threshold-free cluster enhancement option. From each significant cluster that survived correction, FA values were extracted in order to explore the correlations between FA and the physiological parameters.

In order to confirm the validity of TBSS, voxel-based analysis (VBA) [27] of FA images was also conducted using SPM8 at *P*<0.05 at a cluster size of >100 voxels after correcting for FDR.

### Statistical analysis

Demographic and physiological data were analyzed using the Statistical Package for the Social Sciences (SPSS) version 13.0 for Windows (SPSS Inc, Chicago, IL, USA). Differences in the characteristics of NW and OO groups were examined by two-tailed independent sample *t*-test and Chi-square test.

Correlations were detected using the partial correlation model between BMI and plasma glucose level, triglyceride level, HDL level, LDL level, and total cholesterol, controlling for age and gender. FA values and blood parameters were also subjected to partial correlation analysis, with age, gender, and BMI as covariates. *P*<0.05 was considered statistically significant.

## Results

### Demographic and physiological data

The demographic and physiological characteristics of the participants are summarized and demonstrated in Table 1. No significant differences were observed between the NW and OO groups in terms of age, gender, HADS, fasting glucose, or plasma HDL levels. As expected, participants from the NW group had significantly lower BMI (*t = *−12.73, *P*<0.001), smaller waist circumference (*t* = −9.25, *P*<0.001), lower triglyceride levels (*t* = −4.33, *P*<0.001), lower total cholesterol levels (*t* = −2.67, *P*<0.05), and lower LDL levels (*t* = −2.80, *P*<0.05) than those from the OO group. Male and female participants did not differ in terms of BMI, waist circumference, fasting glucose, total cholesterol levels, or plasma HDL levels (Table S1). However, male participants exhibited higher triglyceride (*t* = 3.20, *P* = 0.003) and LDL levels (*t* = 2.03, *P* = 0.048). Further partial correlation analysis showed significant positive correlations between BMI and triglyceride (*r* = 0.523, *P*<0.001), total cholesterol (*r* = 0.426, *P* = 0.003), and LDL levels (*r* = 0.471, *P* = 0.001).

**Table 1:** Demographic and physiological characteristics of participants.

	NW group	OO group	
	(n = 27, Mean ±SD)	(n = 22, Mean ±SD)	*P* value
Age (years)	29.04±7.32	31.72±8.04	0.227
Sex (female/male)	12/15	10/12	0.944
HADS	3.22±1.76	3.77±1.97	0.286
BMI (kg/m^2^)	21.54±2.06	31.44±3.34	**<0.001**
Waist circumference (cm)	76.91±7.70	100.25±9.97	**<0.001**
Fasting glucose (mmol/L)	4.83±0.47	5.07±0.55	0.096
Triglyceride (mmol/L)	1.04±0.39	1.82±0.83	**<0.001**
Total cholesterol (mmol/L)	4.53±0.74	5.03±0.98	**0.010**
LDL-cholesterol (mmol/L)	2.65±0.64	3.28±0.92	**0.007**
HDL-cholesterol (mmol/L)	1.34±0.29	1.19±0.26	0.078

NW: normal weight; OO: obese or overweight; HADS: hospital anxiety and depression scale; BMI: body mass index; LDL: low-density lipoprotein; HDL: high-density lipoprotein.

### VBM analysis

**Figure 1:**
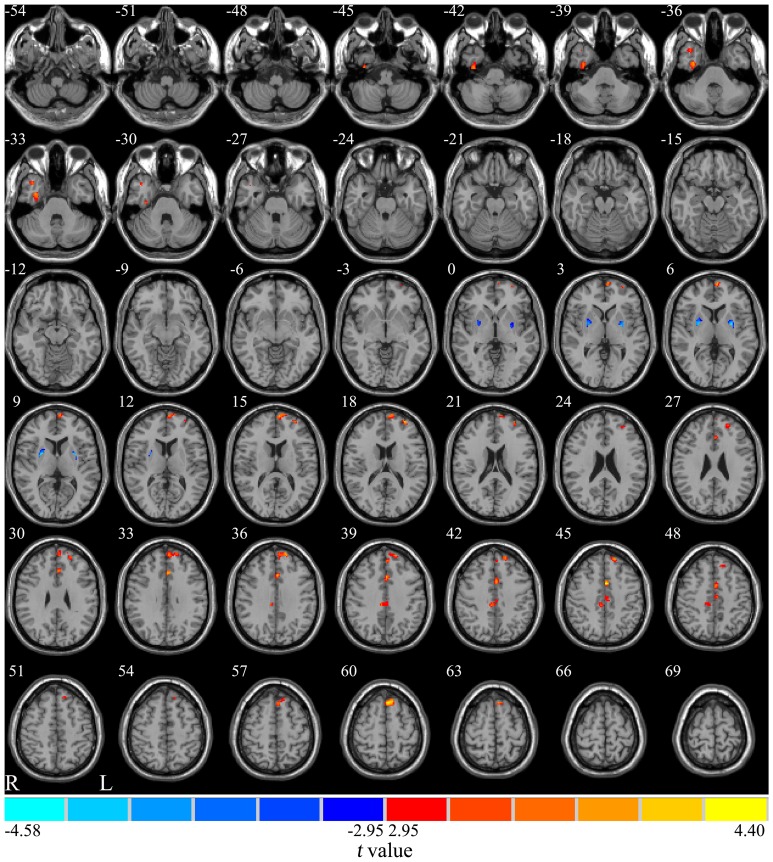
Alteration of GM volume in the OO group compared with the NW group. Red represents regions with reduced GM volume while blue represents those with increased GM volume in the OO group (*P*<0.001, not corrected for multiple comparisons). GM: gray matter; OO: obese or overweight; NW: normal weight.

As shown in Figure 1, compared to the NW group, the OO group demonstrated reduced GM volume (*P*<0.001, not corrected for multiple comparisons) in the right temporal lobe, left prefrontal cortex, and bilateral cingulate gyrus. In addition, the OO group possessed higher GM volume in the bilateral putamen (*P*<0.001, not corrected for multiple comparisons).

**Figure 2:**
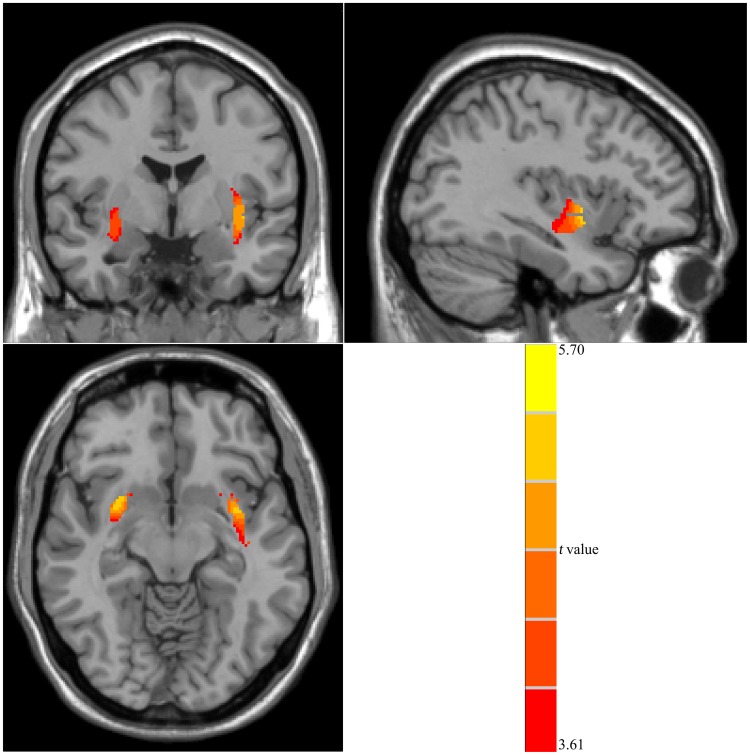
Alteration of WM volume in the OO group compared with the NW group. Red represents regions with significantly reduced WM volume in the OO group (*P*<0.05, FDR corrected). WM: white matter; OO: obese or overweight; NW: normal weight.

As shown in Figure 2, the OO group exhibited significantly smaller volume of WM in the bilateral basal ganglia, the right amygdala, and the left insula (*P*<0.05, FDR corrected). Moreover, compared with the NW group, no brain regions with abnormally large WM volume were observed in the OO group.

### TBSS analysis

**Figure 3:**
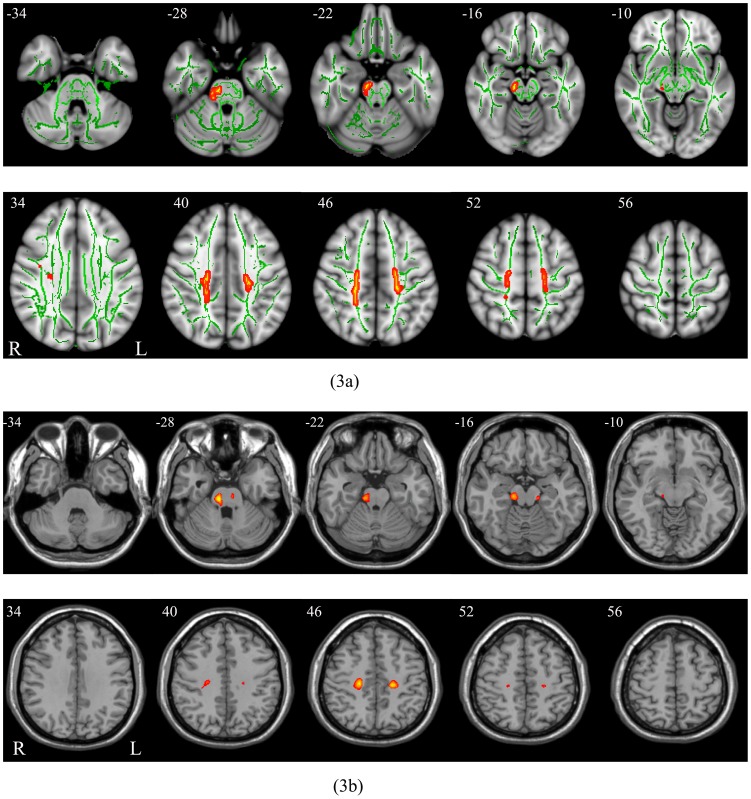
Differences in FA between the OO group and the NW group using both TBSS analysis (Figure 3a) and VBA method (Figure 3b). (**3a**) Green represents mean FA skeleton of all participants, while red represents regions with significantly decreased FA in the OO group (*P*<0.05, corrected for multiple comparisons). (**3b**) Red represents regions with significantly decreased FA in the OO group (*P*<0.05, FDR corrected). OO: obese or overweight; NW: normal weight; FA: fractional anisotropy; TBSS: tract-based spatial statistics; VBA: voxel-based analysis.

Compared to the NW group, the OO group showed significantly lower FA values in the bilateral frontal corticospinal tracts and in the right brainstem according to TBSS (Figure 3a; *P*<0.05, corrected for multiple comparisons) and VBA analyses (Figure 3b; *P*<0.05, FDR corrected).

### Correlation analysis

**Figure 4:**
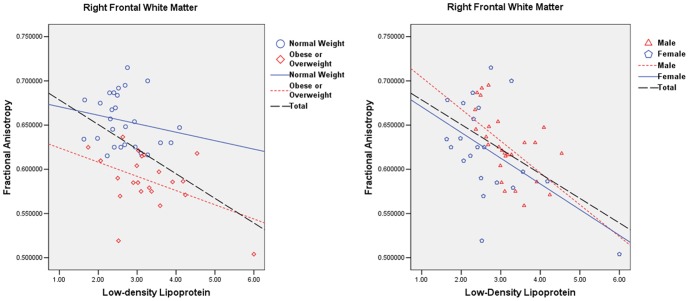
Correlation analysis between plasma LDL levels and FA values extracted from the right frontal corticospinal tract (*P*<0.05). FA values of the right frontal corticospinal tract and plasma LDL levels were significantly correlated in the OO group, rather than the NW group. FA values among male participants showed significant negative correlation with plasma LDL levels. OO: obese or overweight; NW: normal weight; FA: fractional anisotropy; LDL: low-density lipoprotein.

FA values were extracted from three significant clusters reported by TBSS analysis, which included the right frontal corticospinal tract (MNI coordinates: X = 21, Y = −19, Z = 44), the left frontal corticospinal tract (MNI coordinates: X = −21, Y = −21, Z = 43), and corticospinal tract in the right brainstem (MNI coordinates: X = 11, Y = −20, Z = −20). Significant negative correlations were observed between FA values of those three clusters and BMI (*r* = −0.717, *P*<0.001; *r* = −0.528, *P*<0.001; *r* = −0.429, *P* = 0.003) and waist circumference (*r* = −0.676, *P*<0.001; *r* = −0.475, *P* = 0.001; *r* = −0.407, *P* = 0.004). In addition, significant negative correlation was observed between FA values of the right frontal corticospinal tract and plasma LDL (*r* = −0.310, *P* = 0.036) after correcting for age, gender, and BMI (Figure 4). The analysis also revealed that only FA values among male participants showed significant negative correlation with plasma LDL levels (male: *r* = −0.459, *P* = 0.021; female: *r* = −0.146, *P* = 0.539), as shown in Figure 4. Further, partial correlation analyses were performed between FA values and blood parameters separately for the OO and NW groups. For the NW group, no significant correlation was observed between FA values of the three clusters and blood parameters. However, in the OO group, FA values of the right frontal corticospinal tract were significantly correlated with plasma LDL levels (Figure 4; *r* = −0.494, *P* = 0.032).

**Figure 5:**
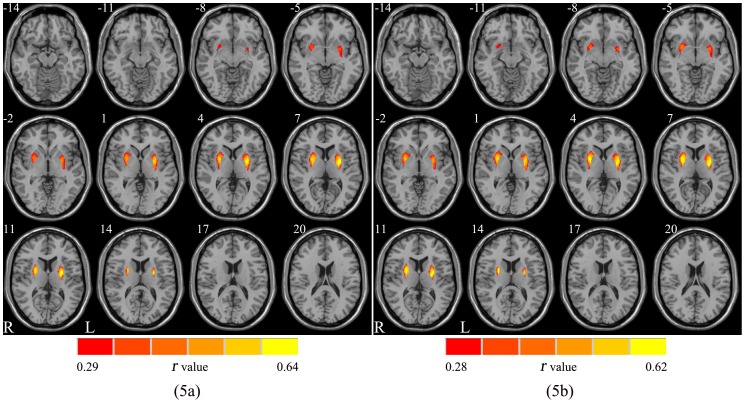
Positive correlation between the volume of bilateral putamen and BMI (5a) and waist circumference (5b) (*P*<0.05, FDR corrected). BMI: body mass index.

The mask of the bilateral putamen was defined using the WFU Pickatlas software (http://www.fmri.wfubmc.edu). After controlling for age and gender, the correlations between the volume of the bilateral putamen and physiological characteristics were examined using the Resting-State fMRI Data Analysis Tookit (REST 2.0; http://resting-fmri.sourceforge.net/). *P*<0.05 was considered statistically significant after correcting for FDR. Two significant correlations were observed between the volume of bilateral putamen and BMI, waist circumference respectively (Figure 5).

## Discussion

The results of this study indicated that healthy young Chinese OO adults had higher BMI, waist circumference, and plasma triglyceride, total cholesterol, and LDL levels compared to their NW counterparts, a finding consistent with the findings of previous studies [28]. Although BMI is the most widely used measurement of body fat, waist circumference is a better indicator of visceral fat and the adverse metabolic effects of obesity; both BMI and waist circumference were measured in this study. The FA values of bilateral frontal corticospinal tracts and the right brainstem were negatively correlated with BMI and waist circumference, while the volume of the bilateral putamen showed positive correlations with both BMI and waist circumference. Further analysis showed that reduced FA values in the right frontal corticospinal tract were significantly correlated with high plasma LDL levels, and that the negative correlation was found only among male participants. To the best of our knowledge, this is the first reported study that evaluates the relationship between plasma LDL and the microstructural impairment in WM in Chinese young adults.

In the current study, VBM with DARTEL was applied to analyze the volumes of GM and WM due to the higher accuracy of this method in spatial normalization than that of traditional VBM. Another recent study in Chinese young adults reported negative correlation between BMI and GM volumes in the midcingulate cortex, the left orbital frontal cortex, and the left ventromedial prefrontal cortex [29]. In our study, GM atrophy was observed in the left prefrontal cortex, the bilateral cingulate gyrus (mainly midcingulate gyrus), and the right temporal lobe in the OO group, a finding that was consistent with the findings of several previously reported studies [10], [12], [29]. Considerable functional studies have shown that prefrontal regions of the brain, particularly, the ventromedial prefrontal cortex (VMPFC) and the dorsolateral prefrontal cortex (DLPFC), along with the anterior and middle cingulate gyrus, are involved in the top-down cognitive control of diet [30], [31]. The control of eating impulse depends on the cingulate gyrus and the interaction between the inhibition regions (DLPFC) and reward value coding regions (VMPFC) [32]. GM atrophy in the left prefrontal regions may suggest less top-down influence of the higher control regions on the lower subcortical reward regions [29], which might be related to bilateral putamen enlargement.

The BMI-related enlarged GM volume in the bilateral putamen observed in our study has not been described in any previous study on obesity. One study among elderly obese individuals showed increased amygdala and hippocampal volumes, but no significant differences in the volumes of the bilateral putamen and globus pallidus [33]. Nevertheless, another study demonstrated that obese individuals exhibited reduced GM volume in the putamen compared to their NW counterparts [10]. The functional brain anatomy and the results of certain other studies would explain the inconsistency of the results of the present study with those of previous studies. Increased activation in the putamen was observed in the obese group, which correlated with the reward function of the putamen in food regulation [34]. The bilateral putamen is highly enriched in dopaminergic neurons, which is essential for the hedonic control of food intake [35]. Given that increased regional brain activation has been demonstrated to be associated with increased GM volume [36], the putamen enlargement observed in this study was considered plausible and might suggest a significant role of food reward in the development of obesity among young adults. It was reported that higher plasma triglyceride and lower plasma high-density lipoprotein (HDL) could account for the positive association between midlife BMI and myo-inositol/creatine in the occipito-parietal GM [37], indicating that abnormal levels of plasma lipids could affect GM volumes. In the present study, we also performed correlation analysis between the bilateral putamen volume and plasma LDL or HDL levels, with age, gender, and BMI as covariates. However, the correlation was not statistically significant after correcting for FDR.

With respect to WM alterations, VBM results demonstrated a widespread reduction of WM volume in the OO group. A recent study by Karlsson et al also revealed broad reduction in WM volume in obese individuals [16]. Although WM reduction in the obese group was observed in both studies, the distribution of the affected WM was slightly different. The reduced WM volume in our study primarily involved regions that regulate food reward, especially, the motivational and incentive properties regulated by the striatum, amygdala, and insula [38]. In contrast, reduced WM volume was also observed in the postcentral somatosensory and occipital visual-processing regions in the obese group in Karlsson et al's research. The differences might be attributed to the different BMI levels of the obese participants in the two studies, which were higher in Karlsson et al's study. Despite these differences, a significant reduction of WM volume in the food reward region was observed in the current study and Karlsson et al's study, suggesting that the OO group might have poor homeostatic regulation of food intake.

Previous studies on the WM integrity of OO individuals mainly adopted the VBA method [16] or ROI-based TBSS analysis [17], [19], while whole-brain, voxel-wise analysis was used in the present study, which combined the TBSS analysis and VBA method. TBSS could complement the limitations of VBA caused by inaccurate alignment of FA images and the problem of smooth kernels, whereas VBA could complement the limitations of TBSS on demonstrating the differences in the peripheral tracts. Thus, TBSS combined with VBA could potentially generate more accurate results of DTI analysis. Both TBSS analysis and VBA method showed that the OO group had lower FA in the bilateral frontal corticospinal tracts and right brainstem in the present study. A recent study by He et al, which applied whole-brain TBSS analysis, found negative correlations between BMI and FA values of bilateral cingulum in Chinese young adults, which also demonstrated obesity-associated microstructural impairment in WM [29]. Furthermore, another study revealed that the FA value of the midcingulate segment positively related to aerobic fitness, which suggests that a sedentary lifestyle may correlate with poor WM integrity [39]. Since the bilateral frontal corticospinal tracts and the right brainstem mainly govern motor function, the present study suggests that the OO individuals may suffer from poor motor control and coordination; however, further studies are required to confirm this hypothesis.

The physiological reasons accounting for obesity-associated impairment of WM integrity are complex. For instance, a high proportion of cholesterol in the peripheral circulation would increase blood-brain barrier (BBB) permeability through a pro-inflammatory pathway [40], thereby affecting the brain cholesterol metabolism [41], although a normal and intact BBB would prevent plasma cholesterol from brain cholesterol. Abnormal brain cholesterol homeostasis contributes to neuronal degeneration and loss of signal transmission, with axonal degeneration constituting one reason for impairment of WM integrity [42], [43]. In the current study, FA values of the bilateral frontal corticospinal tracts and the right brainstem were observed to be negatively correlated with BMI and waist circumference. Further, FA values of the right frontal WM were negatively associated with plasma LDL levels in male participants. A previous study by Cohen et al also reported negative correlation between obesity-related abnormal plasma cholesterols (high LDL, low HDL) and FA in prefrontal regions of the brain [44]. Both Cohen et al's study and the present study suggest that elevated LDL levels partially contribute to obesity-associated impairment in WM integrity. Estrogen-mediated neuroprotection in young females may explain the null result of correlation between FA and plasma LDL in the females [45]. Nevertheless, larger-scale studies to further confirm the correlation between obesity-associated FA modifications and higher triglyceride and cholesterol levels are warranted.

This study had some limitations. First, the difference of GM volume between the OO and NW groups was uncorrected for multiple comparisons, which indicated minor alterations. Second, the present study demonstrated that high plasma LDL levels were solely associated with reduced FA values of the right frontal WM. The observed discrepancies between the results of the present study and those of the previous studies might be attributable to the study design and the relatively limited sample size in our study. This study was conducted in young Han Chinese adults who regularly underwent annual physical examinations and had no indications of diabetes, hyperglycemia, or hypertension. Although the plasma LDL levels in the OO group were significantly higher than those in the NW group, the mean plasma LDL level in the OO group (3.28±0.92 mmol/L) was lower than the LDL level defined as dyslipidemia (≥3.37 mmol/L) [46]. The alteration of GM volume might be a chronic process and affected by combined multiple metabolic factors including diabetes, dyslipidemia, and hypertension [13]. Considering the strict exclusion criteria and the relatively small sample size, our findings suggest early-phase alteration of obesity-related brain structure in young adults and that even a minor elevation of plasma LDL might affect the microstructure of WM. Considering the minor changes in global GM volume, it was possible that the null finding of correlation between plasma LDL and regional GM volume (bilateral putamen) was observed.

## Conclusions

The present study investigated the structural differences in GM and WM between OO and NW Chinese young adults. Although the apparent alteration in GM volume between the OO and NW groups was minor, reduced WM volume and impairment of WM integrity in the OO group were statistically significant. Enlarged bilateral putamen and reduced FA values in the bilateral frontal corticospinal tracts and the right brainstem were significantly associated with high BMI and waist circumference. In addition, the early elevated plasma LDL levels might affect the WM integrity in the right frontal region. Interestingly, this effect was limited to male participants. However, considering the relatively limited amount of participants and the cross-sectional property of this study, further studies with larger sample sizes as well as longitudinal studies are required.

## Supporting Information

Table S1 Demographic and physiological characteristics of participants in different gender.
(DOC)

## References

[1] Finucane MM, Stevens GA, Cowan MJ, Danaei G, Lin JK, et al (2011) National, regional, and global trends in body-mass index since 1980: systematic analysis of health examination surveys and epidemiological studies with 960 country-years and 9.1 million participants. Lancet 377: 557–567. 21295846 10.1016/S0140-6736(10)62037-5 PMC4472365

[2] Li XY, Jiang Y, Hu N, Li YC, Zhang M, et al (2012) [Prevalence and characteristic of overweight and obesity among adults in China, 2010]. Zhonghua Yu Fang Yi Xue Za Zhi 46: 683–686. 23157859

[3] Wang H, Du S, Zhai F, Popkin BM (2007) Trends in the distribution of body mass index among Chinese adults, aged 20–45 years (1989–2000). Int J Obes (Lond) 31: 272–278. 16788569 10.1038/sj.ijo.0803416

[4] Wang H, Zhai F (2013) Programme and policy options for preventing obesity in China. Obes Rev 14 Suppl 2134–140. 24102781 10.1111/obr.12106 PMC4048452

[5] Vance JE (2012) Dysregulation of cholesterol balance in the brain: contribution to neurodegenerative diseases. Disease models & mechanisms 5: 746–755. 23065638 10.1242/dmm.010124 PMC3484857

[6] Umeda T, Tomiyama T, Kitajima E, Idomoto T, Nomura S, et al (2012) Hypercholesterolemia accelerates intraneuronal accumulation of Abeta oligomers resulting in memory impairment in Alzheimer's disease model mice. Life Sci 91: 1169–1176. 22273754 10.1016/j.lfs.2011.12.022

[7] Shepardson NE, Shankar GM, Selkoe DJ (2011) Cholesterol level and statin use in Alzheimer disease: I. Review of epidemiological and preclinical studies. Archives of neurology 68: 1239–1244. 21987540 10.1001/archneurol.2011.203 PMC3211071

[8] Walther K, Birdsill AC, Glisky EL, Ryan L (2010) Structural brain differences and cognitive functioning related to body mass index in older females. Hum Brain Mapp 31: 1052–1064. 19998366 10.1002/hbm.20916 PMC6870943

[9] Whitmer RA, Gunderson EP, Barrett-Connor E, Quesenberry CP Jr, Yaffe K (2005) Obesity in middle age and future risk of dementia: a 27 year longitudinal population based study. BMJ 330: 1360. 15863436 10.1136/bmj.38446.466238.E0 PMC558283

[10] Pannacciulli N, Del Parigi A, Chen K, Le DS, Reiman EM, et al (2006) Brain abnormalities in human obesity: a voxel-based morphometric study. Neuroimage 31: 1419–1425. 16545583 10.1016/j.neuroimage.2006.01.047

[11] Bobb JF, Schwartz BS, Davatzikos C, Caffo B (2012) Cross-sectional and longitudinal association of body mass index and brain volume. Hum Brain Mapp. 10.1002/hbm.22159 PMC3615109 23008165

[12] Taki Y, Kinomura S, Sato K, Inoue K, Goto R, et al (2008) Relationship between body mass index and gray matter volume in 1,428 healthy individuals. Obesity (Silver Spring) 16: 119–124. 18223623 10.1038/oby.2007.4

[13] Yokum S, Ng J, Stice E (2012) Relation of regional gray and white matter volumes to current BMI and future increases in BMI: a prospective MRI study. Int J Obes (Lond) 36: 656–664. 21894161 10.1038/ijo.2011.175 PMC3982917

[14] Haltia LT, Viljanen A, Parkkola R, Kemppainen N, Rinne JO, et al (2007) Brain white matter expansion in human obesity and the recovering effect of dieting. J Clin Endocrinol Metab 92: 3278–3284. 17536002 10.1210/jc.2006-2495

[15] Raji CA, Ho AJ, Parikshak NN, Becker JT, Lopez OL, et al (2010) Brain structure and obesity. Hum Brain Mapp 31: 353–364. 19662657 10.1002/hbm.20870 PMC2826530

[16] Karlsson HK, Tuulari JJ, Hirvonen J, Lepomaki V, Parkkola R, et al. (2013) Obesity is associated with white matter atrophy: A combined diffusion tensor imaging and voxel-based morphometric study. Obesity. 10.1002/oby.20386 23512884

[17] Stanek KM, Grieve SM, Brickman AM, Korgaonkar MS, Paul RH, et al (2011) Obesity is associated with reduced white matter integrity in otherwise healthy adults. Obesity (Silver Spring) 19: 500–504. 21183934 10.1038/oby.2010.312

[18] Verstynen TD, Weinstein AM, Schneider WW, Jakicic JM, Rofey DL, et al (2012) Increased body mass index is associated with a global and distributed decrease in white matter microstructural integrity. Psychosom Med 74: 682–690. 22879428 10.1097/PSY.0b013e318261909c PMC3586991

[19] Mueller K, Anwander A, Moller HE, Horstmann A, Lepsien J, et al (2011) Sex-dependent influences of obesity on cerebral white matter investigated by diffusion-tensor imaging. Plos One 6: e18544. 21494606 10.1371/journal.pone.0018544 PMC3073967

[20] Wang W, Wang K, Li T, Xiang H, Ma L, et al (2002) [A discussion on utility and purposed value of obesity and abdomen obesity when body mass index, waist circumference, waist to hip ratio used as indexes predicting hypertension and hyper-blood glucose]. Zhonghua Liu Xing Bing Xue Za Zhi 23: 16–19. 12015102

[21] Herrero MJ, Blanch J, Peri JM, De Pablo J, Pintor L, et al (2003) A validation study of the hospital anxiety and depression scale (HADS) in a Spanish population. General hospital psychiatry 25: 277–283. 12850660 10.1016/s0163-8343(03)00043-4

[22] Ashburner J (2007) A fast diffeomorphic image registration algorithm. Neuroimage 38: 95–113. 17761438 10.1016/j.neuroimage.2007.07.007

[23] Klein A, Andersson J, Ardekani BA, Ashburner J, Avants B, et al (2009) Evaluation of 14 nonlinear deformation algorithms applied to human brain MRI registration. Neuroimage 46: 786–802. 19195496 10.1016/j.neuroimage.2008.12.037 PMC2747506

[24] Smith SM, Jenkinson M, Woolrich MW, Beckmann CF, Behrens TE, et al (2004) Advances in functional and structural MR image analysis and implementation as FSL. Neuroimage 23 Suppl 1S208–219. 15501092 10.1016/j.neuroimage.2004.07.051

[25] Smith SM, Jenkinson M, Johansen-Berg H, Rueckert D, Nichols TE, et al (2006) Tract-based spatial statistics: voxelwise analysis of multi-subject diffusion data. Neuroimage 31: 1487–1505. 16624579 10.1016/j.neuroimage.2006.02.024

[26] Rueckert D, Sonoda LI, Hayes C, Hill DL, Leach MO, et al (1999) Nonrigid registration using free-form deformations: application to breast MR images. IEEE Trans Med Imaging 18: 712–721. 10534053 10.1109/42.796284

[27] Ashburner J, Friston KJ (2000) Voxel-based morphometry–the methods. Neuroimage 11: 805–821. 10860804 10.1006/nimg.2000.0582

[28] Hou X, Lu J, Weng J, Ji L, Shan Z, et al (2013) Impact of waist circumference and body mass index on risk of cardiometabolic disorder and cardiovascular disease in Chinese adults: a national diabetes and metabolic disorders survey. Plos One 8: e57319. 23520466 10.1371/journal.pone.0057319 PMC3592870

[29] He Q, Chen C, Dong Q, Xue G, Lu ZL, et al. (2013) Gray and white matter structures in the midcingulate cortex region contribute to body mass index in Chinese young adults. Brain structure & function. 10.1007/s00429-013-0657-9 PMC3995892 24146133

[30] Stice E, Figlewicz DP, Gosnell BA, Levine AS, Pratt WE (2013) The contribution of brain reward circuits to the obesity epidemic. Neuroscience and biobehavioral reviews 37: 2047–2058. 23237885 10.1016/j.neubiorev.2012.12.001 PMC3604128

[31] Shackman AJ, Salomons TV, Slagter HA, Fox AS, Winter JJ, et al (2011) The integration of negative affect, pain and cognitive control in the cingulate cortex. Nature reviews Neuroscience 12: 154–167. 21331082 10.1038/nrn2994 PMC3044650

[32] Weygandt M, Mai K, Dommes E, Leupelt V, Hackmack K, et al (2013) The role of neural impulse control mechanisms for dietary success in obesity. Neuroimage 83: 669–678. 23867558 10.1016/j.neuroimage.2013.07.028

[33] Widya RL, de Roos A, Trompet S, de Craen AJ, Westendorp RG, et al (2011) Increased amygdalar and hippocampal volumes in elderly obese individuals with or at risk of cardiovascular disease. Am J Clin Nutr 93: 1190–1195. 21450935 10.3945/ajcn.110.006304

[34] Geliebter A (2013) Neuroimaging of gastric distension and gastric bypass surgery. Appetite. 10.1016/j.appet.2013.07.002 PMC3919638 23932915

[35] Volkow ND, Wang GJ, Baler RD (2011) Reward, dopamine and the control of food intake: implications for obesity. Trends in cognitive sciences 15: 37–46. 21109477 10.1016/j.tics.2010.11.001 PMC3124340

[36] Brodtmann A, Puce A, Darby D, Donnan G (2009) Regional fMRI brain activation does correlate with global brain volume. Brain Res 1259: 17–25. 19133239 10.1016/j.brainres.2008.12.044

[37] Haley AP, Gonzales MM, Tarumi T, Tanaka H (2013) Dyslipidemia links obesity to early cerebral neurochemical alterations. Obesity (Silver Spring). 10.1002/oby.20332 PMC3695042 23512296

[38] Nummenmaa L, Hirvonen J, Hannukainen JC, Immonen H, Lindroos MM, et al (2012) Dorsal striatum and its limbic connectivity mediate abnormal anticipatory reward processing in obesity. Plos One 7: e31089. 22319604 10.1371/journal.pone.0031089 PMC3272045

[39] Marks BL, Katz LM, Styner M, Smith JK (2011) Aerobic fitness and obesity: relationship to cerebral white matter integrity in the brain of active and sedentary older adults. British journal of sports medicine 45: 1208–1215. 20558529 10.1136/bjsm.2009.068114

[40] Ehrlich D, Humpel C (2012) Chronic vascular risk factors (cholesterol, homocysteine, ethanol) impair spatial memory, decline cholinergic neurons and induce blood-brain barrier leakage in rats in vivo. J Neurol Sci 322: 92–95. 22819352 10.1016/j.jns.2012.07.002 PMC3484398

[41] Hui L, Chen X, Geiger JD (2012) Endolysosome involvement in LDL cholesterol-induced Alzheimer's disease-like pathology in primary cultured neurons. Life Sci 91: 1159–1168. 22580286 10.1016/j.lfs.2012.04.039 PMC3431446

[42] Hung YH, Bush AI, La Fontaine S (2013) Links between copper and cholesterol in Alzheimer's disease. Frontiers in physiology 4: 111. 23720634 10.3389/fphys.2013.00111 PMC3655288

[43] Aung WY, Mar S, Benzinger TL (2013) Diffusion tensor MRI as a biomarker in axonal and myelin damage. Imaging in medicine 5: 427–440. 24795779 10.2217/iim.13.49 PMC4004089

[44] Cohen JI, Cazettes F, Convit A (2011) Abnormal cholesterol is associated with prefrontal white matter abnormalities among obese adults: A diffusion tensor imaging study. Neuroradiology Journal 24: 854–861. 24059886 10.1177/197140091102400604

[45] Peri A, Benvenuti S, Luciani P, Deledda C, Cellai I (2011) Membrane cholesterol as a mediator of the neuroprotective effects of estrogens. Neuroscience 191: 107–117. 21396986 10.1016/j.neuroscience.2011.03.011

[46] Third Report of the National Cholesterol Education Program (NCEP) Expert Panel on Detection, Evaluation, and Treatment of High Blood Cholesterol in Adults (Adult Treatment Panel III) final report. Circulation 106: 3143–3421. 12485966

