# Supplementary material for: Reduced Right Frontal Fractional Anisotropy Correlated with Early Elevated Plasma LDL Levels in Obese Young Adults
Source: PLoS One. 2014 Oct 3;9(10):e108180. doi: 10.1371/journal.pone.0108180 (PMC4184805; doi:10.1371/journal.pone.0108180)
Supplement: Table S1 — Demographic and physiological characteristics of participants in different gender. (DOC) [file pone.0108180.s001.doc]

**Table S1. Demographic and physiological characteristics of participants in different gender.**

|  | Male | Female |  |
| --- | --- | --- | --- |
|  | (n=27, Mean±SD) | (n=22, Mean±SD) | *P* value |
| Age (years) | 30.74±8.11 | 29.64±7.29 | 0.622 |
| HADS | 3.00±1.36 | 4.04±2.24 | 0.063 |
| BMI (kg/m^2^) | 26.65±5.75 | 25.17±5.54 | 0.366 |
| Waist circumference (cm) | 90.89±15.36 | 83.09±12.66 | 0.062 |
| Fasting glucose (mmol/L) | 5.06±0.60 | 4.79±0.35 | 0.067 |
| Triglyceride (mmol/L) | 1.65±0.82 | 1.07±0.43 | **0.003** |
| Total cholesterol (mmol/L) | 5.05±0.70 | 4.56±1.08 | 0.063 |
| LDL-cholesterol (mmol/L) | 3.14±0.60 | 2.68±0.97 | **0.048** |
| HDL-cholesterol (mmol/L) | 1.21±0.31 | 1.35±0.24 | 0.099 |

HADS: hospital anxiety and depression scale; BMI: body mass index; LDL: low-density lipoprotein; HDL: high-density lipoprotein.
